# Automatically Appraising the Credibility of Vaccine-Related Web Pages Shared on Social Media: A Twitter Surveillance Study

**DOI:** 10.2196/14007

**Published:** 2019-11-04

**Authors:** Zubair Shah, Didi Surian, Amalie Dyda, Enrico Coiera, Kenneth D Mandl, Adam G Dunn

**Affiliations:** 1 Centre for Health Informatics Australian Institute of Health Innovation Macquarie University Sydney Australia; 2 Division of Information and Communication Technology, College of Science and Engineering Hamad Bin Khalifa University Doha Qatar; 3 Department of Biomedical Informatics, Harvard Medical School Boston, MA United States; 4 Computational Health Informatics Program, Boston Children’s Hospital Boston, MA United States

**Keywords:** health misinformation, credibility appraisal, machine learning, social media

## Abstract

**Background:**

Tools used to appraise the credibility of health information are time-consuming to apply and require context-specific expertise, limiting their use for quickly identifying and mitigating the spread of misinformation as it emerges.

**Objective:**

The aim of this study was to estimate the proportion of vaccine-related Twitter posts linked to Web pages of low credibility and measure the potential reach of those posts.

**Methods:**

Sampling from 143,003 unique vaccine-related Web pages shared on Twitter between January 2017 and March 2018, we used a 7-point checklist adapted from validated tools and guidelines to manually appraise the credibility of 474 Web pages. These were used to train several classifiers (random forests, support vector machines, and recurrent neural networks) using the text from a Web page to predict whether the information satisfies each of the 7 criteria. Estimating the credibility of all other Web pages, we used the follower network to estimate potential exposures relative to a credibility score defined by the 7-point checklist.

**Results:**

The best-performing classifiers were able to distinguish between low, medium, and high credibility with an accuracy of 78% and labeled low-credibility Web pages with a precision of over 96%. Across the set of unique Web pages, 11.86% (16,961 of 143,003) were estimated as low credibility and they generated 9.34% (1.64 billion of 17.6 billion) of potential exposures. The 100 most popular links to low credibility Web pages were each potentially seen by an estimated 2 million to 80 million Twitter users globally.

**Conclusions:**

The results indicate that although a small minority of low-credibility Web pages reach a large audience, low-credibility Web pages tend to reach fewer users than other Web pages overall and are more commonly shared within certain subpopulations. An automatic credibility appraisal tool may be useful for finding communities of users at higher risk of exposure to low-credibility vaccine communications.

## Introduction

### Background

The spread of misinformation, which we define here to include communications that are not a fair representation of available evidence or communicate that evidence poorly, has become an increasingly studied topic in various domains [1-8]. Misinformation can cause harm by influencing attitudes and beliefs [9,10]. Although the rapid growth of Web-based communications has benefited public health by providing access to a much broader range of health information, most people trust health information available on the Web without attempting to validate the sources [11,12], despite concerns about the presence of misinformation in what they access [13] and known issues where biases and marketing can lead to the miscommunication of evidence [14-18]. Proposed approaches for mitigating the impact of misinformation include empowering individuals to better deal with the information they encounter and improvements in the automatic detection of misinformation on Web-based platforms [1].

Most studies aimed at finding or tracking misinformation on social media define misinformation using *veracity*—whether a claim is true or false or real or fake. In the health domain, veracity alone often does not provide enough information to be useful in understanding the range of factors that might influence attitudes and behaviors, such as persuasiveness, timeliness, or applicability. The *credibility* of health communications thus includes a broader set of factors that include veracity as well as readability and clarity, the use and transparency of sources, biases and false balance, and disclosure of conflicts of interest [19]. It is important to consider credibility when evaluating the potential impact of health communications on health attitudes and outcomes because certain types of communication can be true but misleading, such as in the case of false balance in news media [20].

A range of tools have been developed to assess the credibility of health information available on the Web. Most were designed as checklists to be used by experts to assess the credibility and transparency of what they are reading. The DISCERN tool was designed as a general purpose tool for evaluating the quality of health information [21], with an emphasis on Web pages that patients might use to support the decisions they make about their health. The Quality Index for health-related Media Reports (QIMR) is a more recent example and differs from previous tools in that it was designed to be used to evaluate the quality of communications about new biomedical research [22]. Common elements of the tools used by experts to assess the credibility of health research reporting and patient information on the Web include the following: the veracity of the included information, transparency about sources of evidence, disclosure of advertising, simplicity and readability of the language, and use of balanced language that does not distort or sensationalize [19]. Most of the tools can be time-consuming to use and often require specific training or expertise to apply. Organizations such as HealthNewsReview that ended in 2018 used experts to evaluate new health-related communications as they appear in the news media [23].

Public perception of vaccines is an exemplar of the problem of misinformation spread through news and social media [24]. Beyond public health and vaccines, previous studies using social media data derived from Twitter to understand the spread and impact of misinformation have variously extracted text from what users post or information about their social connections [25-29]. Attitudes toward vaccines and opinions about disease outbreaks are a common application domain studied in social media research [30-34]. In particular, studies of human papillomavirus (HPV) vaccines have made use of the information users post and their social connections, as well as what people might have been exposed to from their networks [35-38]. The ability to measure how people engage and share misinformation on social media may help us better target and monitor the impact of public health interventions [39-41].

Given the rate at which new information is made available and the resources needed to appraise them, there is currently no way to keep up with new health-related stories as soon as they appear. Although the challenge of managing information volume versus quality was discussed two decades ago [42], methods for managing emerging misinformation in health-related news and media remain an unresolved issue for public health.

### Research Objectives

We sought to characterize the sharing and potential reach of vaccine-related Web pages shared on Twitter, relative to credibility. As it would not have been feasible to manually assess the credibility of all Web pages, we developed and evaluated classifiers to automatically estimate their credibility.

## Methods

### Overview

The study used a retrospective observational design. To estimate the credibility of vaccine-related Web pages shared on Twitter, we collected text from vaccination-related Web pages by monitoring links from tweets that mentioned relevant keywords. We manually appraised the credibility of a sample of Web pages by applying a checklist-based appraisal tool, using the sample to train classifiers to predict a credibility score in unseen Web pages. Applying an ensemble classifier to the full set of Web pages collected as part of the surveillance, we examined patterns of sharing relative to credibility scores.

### Datasets

We collected 6,591,566 English language, vaccine-related tweets and retweets from 1,860,662 unique Twitter users between January 17, 2017, and March 14, 2018, using the Twitter Search Application Programming Interface, using a set of predefined search terms (including “vaccin*,” “immunis*,” “vax*,” and “antivax*”). For all unique users posting vaccine-related tweets during the study period, we collected the lists of their followers to construct the social network.

**Figure 1:**
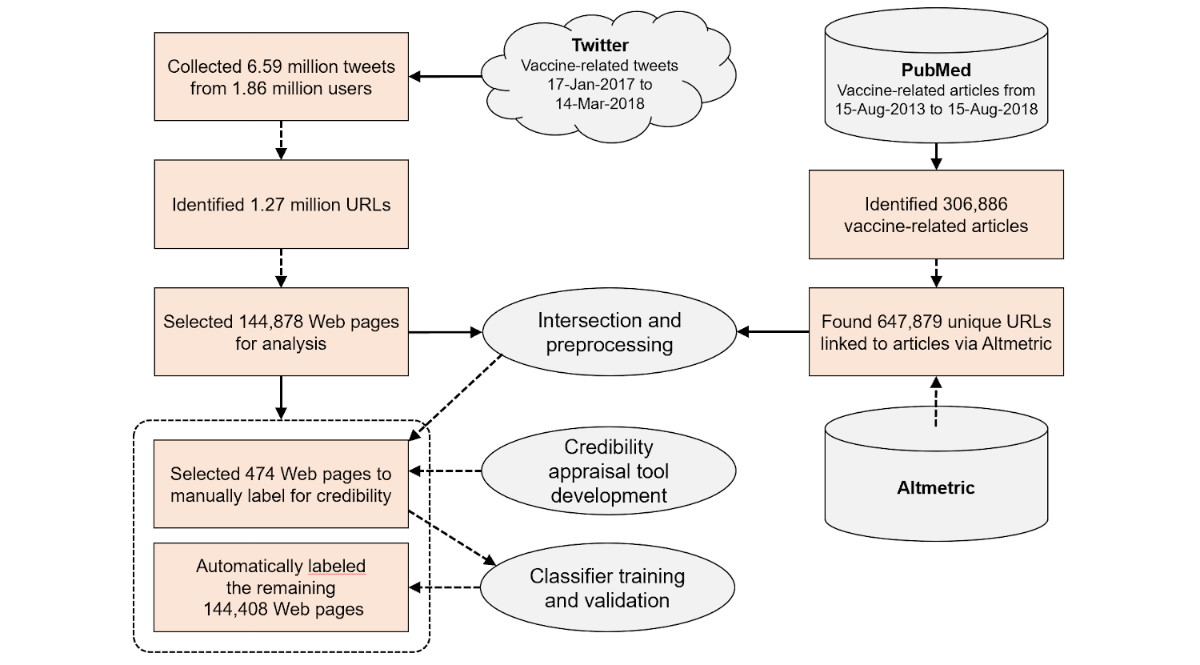
The steps used to define the training dataset and automatically label Web pages.

We extracted 1.27 million unique URLs from the set of tweets to identify the set of text-based Web pages to include in the analysis. To restrict the set of Web pages to only English language text, we used a Google library [43]; removed other Web pages that were internal Twitter links, broken links, or links to Web pages that were no longer available; and removed Web pages with fewer than 300 words in contiguous blocks. We then checked for duplicates of other Web pages already included, removing Web pages for which most of the text was equivalent to another Web page in the set, retaining the Web page with the greatest number of words. The remaining set of 143,003 Web pages (Figure 1) was used in the subsequent analysis.

To modify how we sampled tweets for constructing a manually labeled dataset, we used PubMed to search for vaccine-related research articles using search terms “vaccine” or “immunisation” in the title or abstract, automatically expanded by PubMed to include synonyms and MeSH terms. The search returned 306,886 articles. We then used the PubMed identifiers of these articles with Altmetric (Digital Science) to identify Web pages (news, blogs, and social media posts) that linked to these articles via their digital object identifier, PubMed entry, or journal Web page. We found 647,879 unique URLs from Altmetric that cited the selected vaccines-related PubMed articles.

The intersection of the URLs extracted from Altmetric and the URLs extracted from the tweets allowed us to oversample from the set of Web pages for which we expected to have higher-credibility scores (described below). This approach also allowed us to exclude most of the URLs shared on Twitter that linked directly to research articles by removing the tweets that were identified by Altmetric.

### Credibility Appraisal Tool

The credibility appraisal tool was developed by 3 investigators (AGD, AD, and MS) with expertise in public health, public health informatics, science communication, and journalism. To develop a tool that would work specifically with vaccine-related Web pages, the investigators adapted and synthesized individual criteria from the following checklist-based tools and guidelines [19]:

- Centers for Disease Control and Prevention guide for creating health materials [44]
- The DISCERN tool [21]
- Health News Review criteria [23] that is informed by Moynihan et al [45] and the Statement of Principles of the Association of Health Care Journalists [46]
- Media Doctor review criteria [47]
- World Health Organization report on vaccination and trust [48]
- The QIMR [22].

Using these documents as a guide, we adapted from the DISCERN and QIMR checklists, and added 2 additional criteria that were specific to vaccine-related communications. The tool was pilot tested on 30 randomly selected Web pages and iteratively refined through discussion among the 3 investigators. The resulting credibility appraisal tool included the following 7 criteria: (1) information presented is based on objective, scientific research; (2) adequate detail about the level of evidence offered by the research is included; (3) uncertainties and limitations in the research in focus are described; (4) the information does not exaggerate, overstate, or misrepresent available evidence; (5) provides context for the research in focus; (6) uses clear, nontechnical language that is easy to understand; and (6) is transparent about sponsorship and funding.

### Manually Labeled Sample

**Figure 2:**
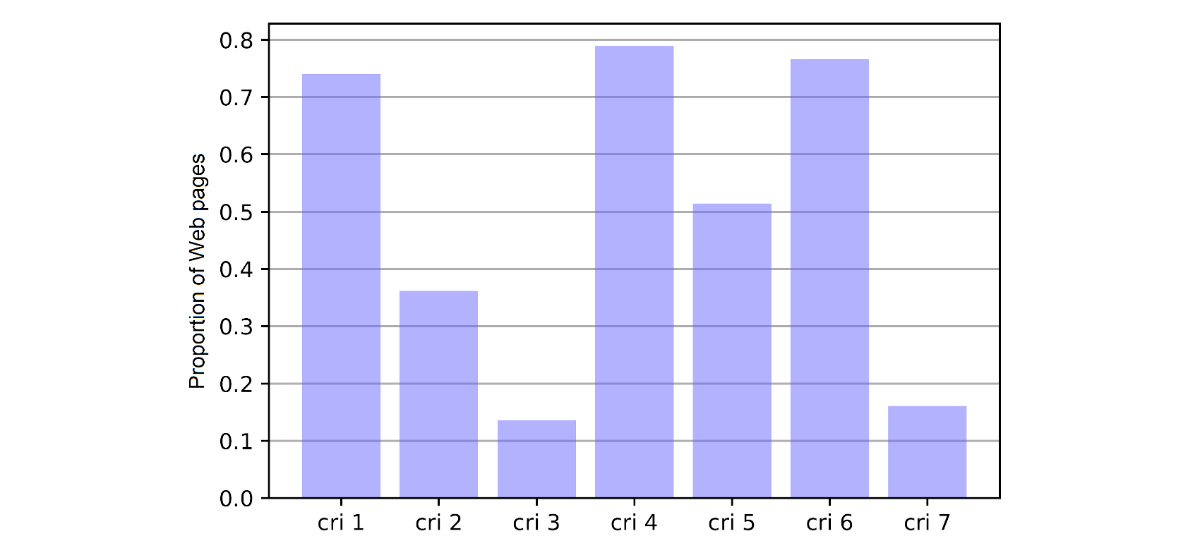
The proportion of Web pages that met the individual criteria in the 474 Web pages used to train the classifiers. cri: criterion.

The 3 investigators then applied the credibility appraisal tool to an additional 474 vaccine-related Web pages. For each Web page, investigators navigated to the website, read the article, and decided whether it satisfied each of the 7 criteria. This process produced a set of values (0 or 1) for each criterion and Web page. We then summarized the information as a *credibility score*, defined by the number of criteria that were satisfied, and grouped Web pages by credibility score into low (0-2 criteria satisfied), medium (3-4 criteria satisfied), and high (5-7 criteria satisfied). Across the 474 expert-labeled examples, the proportion of the Web pages that were judged to have satisfied each of the 7 credibility criteria varied substantially (Figure 2).

The investigators independently undertook duplicate appraisals of a subset of the Web pages to measure inter-rater reliability, and it was found to be reasonable for separating Web pages as low, medium, or high credibility (Fleiss kappa 0.46; 95% CI 0.41-0.52; *P*<.001) and near-perfect when the aim was to separate low-credibility Web pages from all others (Fleiss kappa 0.89; 95% CI 0.82-0.97; *P*<.001). The design of the checklist suggests that it is a useful approach for identifying Web pages of low credibility.

### Classifier Design

We compared 3 machine learning methods that are commonly used for document classification problems: support vector machines (SVM), random forests (RF), and recurrent neural networks (RNN). The SVM method trains a large-margin classifier that aims to find a decision boundary between 2 classes that is maximally far from any point in the training data. In the RF method classification, trees are constructed by randomly selecting a subspace of features at each node of the decision tree to grow branches. The method then uses bagging to generate subsets of training data for constructing individual trees, which are then combined to form RF model. The RNN method refers to a class of artificial neural networks comprising neural network blocks that are linked to each other to form a directed graph along a sequence. The method is used to model dynamic temporal behavior for a time sequence, which is useful for understanding the language.

The aim of these supervised machine learning techniques was to train a model to predict the class of an unseen document by learning how to distinguish the language used across classes. To apply the classifiers, we cleaned the text downloaded from Web pages by removing extra spaces, tabs, extra newlines, and nonstandard characters including emoticons. Each Web page was then included as a document in our corpus.

To develop the RNN classifier, we used average-stochastic gradient descent weight-dropped long short-term memory [49]. In what follows, we refer to this as the deep learning (DL)–based classifier. The DL-based classifier comprised a backbone and custom head. The backbone is a language model that is a deep RNN. The head is a linear classifier comprising 2 linear blocks with rectified linear unit activations for the intermediate layer and a softmax activation for the final layer that can estimate the target labels (in our case, whether it satisfies a credibility criterion).

Language models are trained to understand the structure of the language used in a corpus of documents, and its performance is measured by its ability to predict the next word in a sentence based on the set of previous words. After the language model is trained for this task, the complete DL-based classifier is then fine-tuned to predict whether a document satisfies each of the credibility checklist criteria. Language models are often trained to learn the structure of the language in a target corpus, but recent advances in transfer learning have produced superior results including shorter training times and higher performance. An example is the Universal Language Model Fine-Tuning method [50], which was proposed and evaluated on natural language processing tasks.

**Figure 3:**
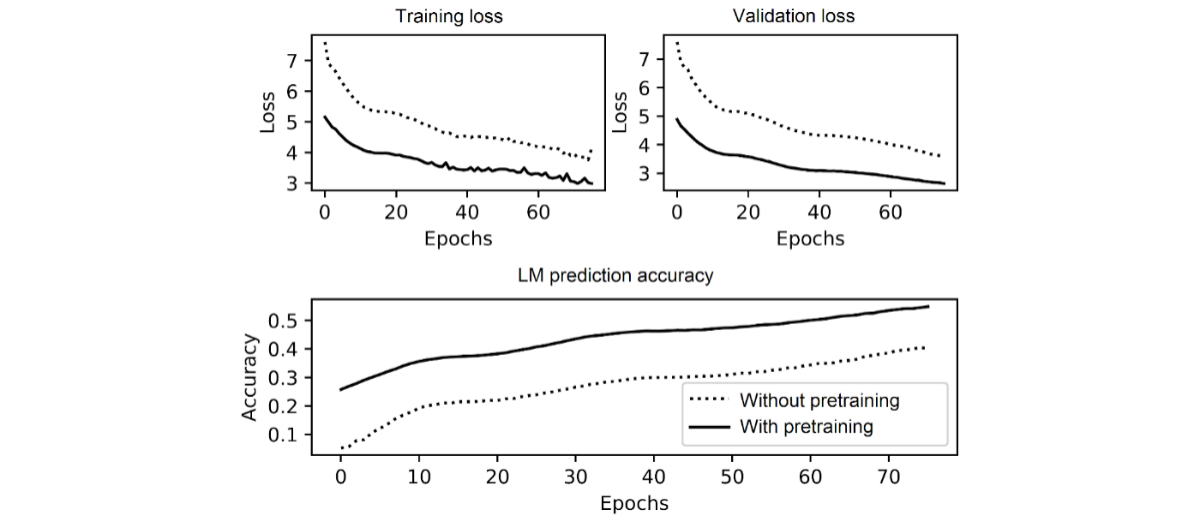
The performance difference of the language model (LM) for 2 different settings, including training loss (top-left), validation cross-entropy loss (top-right), and the accuracy of the LM predicting the next word in a sentence given previous words in the validation text (bottom).

We used transfer learning to create the language model backbone. The language model was developed with 3 layers, 1150 hidden units, and an embedding size of 400 per word, and the weights were initialized from a pretrained WikiText-103 language model produced by Howard et al [50]. The parameters and values used in the initialization of the language model and classifier are given in Table 1. The results of the performance of the associated language model are given in Figure 3.

**Table 1:** The parameters and corresponding values for the initialization of the language model and classifier.

Parameters	Value
Weight decay	1.00E-04
Backpropagation through time	60
Batch size	52
Dropouts	0.25, 0.1, 0.2, 0.02, 0.15
Embedding size	400
Number of layers	3 (language model), 5 (classifier)
Optimizer	Adam
β_1_, β_2_	0.8, 0.99

For the SVM- and RF-based classifiers, we performed additional preprocessing to remove stop words and low-frequency words to improve accuracy. After preprocessing, there were 60,660 unique words used across the entire corpus; these were used as features for training and testing RF and SVM classifiers. Each document was represented as a set of feature vectors, where features were defined by term frequency–inverse document frequency (tf-idf) weights. tf-idf represents the importance of a word to a document in a corpus, which increases proportionally to the number of times it appears in the document but is offset by the frequency of the word in the corpus, ensuring that the similarity between documents be more influenced by discriminative words with relatively low frequencies in the corpus. The best parameters for SVM and RF are found using grid search functionality of *scikit-learn* library and are given in Table 2.

Using the expert-labeled data, we trained 21 classifiers (1 per criterion for each of the RF-, SVM-, and DL-based classifiers) and evaluated the performance of the classifiers in 10-fold cross-validation tests, reporting the average F_1_ score and accuracy for all 3 classifiers. Although the comparison of the performance across the set of classifiers may be of interest, our aim was to provide the basis for an ensemble classifier that could reliably estimate which of the criteria were met by each Web page.

**Table 2:** The parameters used for support vector machine and random forest classifiers; all other parameters are kept as default.

Parameters		Value
**Support vector machines**		
	C	100
	Gamma	1
	Kernel	linear
	Norm	l1
	Use-idf^a^	TRUE
	Max-df^b^	1
	N-gram range	(1,1)
**Random forests**		
	N-estimators	10
	Criterion	Gini
	Min-impurity-split	1.00E-07

^a^Use-idf: when true, term weights are scaled by the number of documents they appear in.

^b^Max-df: when set to 1, words that appear in every document are not removed.

### Sharing and Potential Exposure Estimation

Following the development of a reliable tool for automatically estimating the credibility of vaccine-related communications at scale, we aimed to characterize patterns of potential exposure to low-credibility vaccine communications on Twitter. For each Web page that met our study inclusion criteria, we estimated its credibility score using the best-performing classifiers for each criterion. We then aggregated the total number of tweets posted during the study period that included a link to the Web page, including tweets and retweets. We then estimated the *potential exposure* by summing the total number of followers for all tweets and retweets. Note that this represents the maximum possible audience, and we did not identify the unique set of users who might have been exposed at least once because of who they follow as we had done in previous studies [14].

To examine how users posting links to low-credibility Web pages might be concentrated within or across subpopulations, we also estimated a per-user measure of credibility, which was defined by the list of credibility scores for any user sharing links to one or more Web pages. We used these lists in conjunction with information about followers to construct a *follower network*, which allowed us to identify subpopulations of Twitter users for which the sharing of low-credibility vaccine communications was common.

## Results

### Classifier Performance

The RF classifiers produced the highest performance overall, and in most cases predicted, whether the text on a vaccine-related Web page satisfied each of the credibility criteria with over 90% accuracy (Table 3). The SVM-based classifier produced the highest F_1_ scores for 2 of the most unbalanced criteria. Further experiments are needed to determine whether the DL-based classifier outperforms baseline methods if more expert-labeled data are made available. The results show that it is feasible to estimate credibility appraisal for Web pages about vaccination without additional human input, suggesting the performance—although variable—is high enough to warrant their use in surveillance.

Where the best-performing classifiers were combined to distinguish between low-, medium-, and high-credibility Web pages, the overall accuracy of the ensemble classifier that combines best-performing classifiers (SVM for criterion 3 and 7 and RF for all other criteria) was 78.30%. In terms of labeling low-credibility Web pages, the ensemble classifier rarely mislabeled a high- or medium-credibility Web page as low credibility; more than 19 out of every 20 Web pages labeled as low credibility were correct.

**Figure 4:**
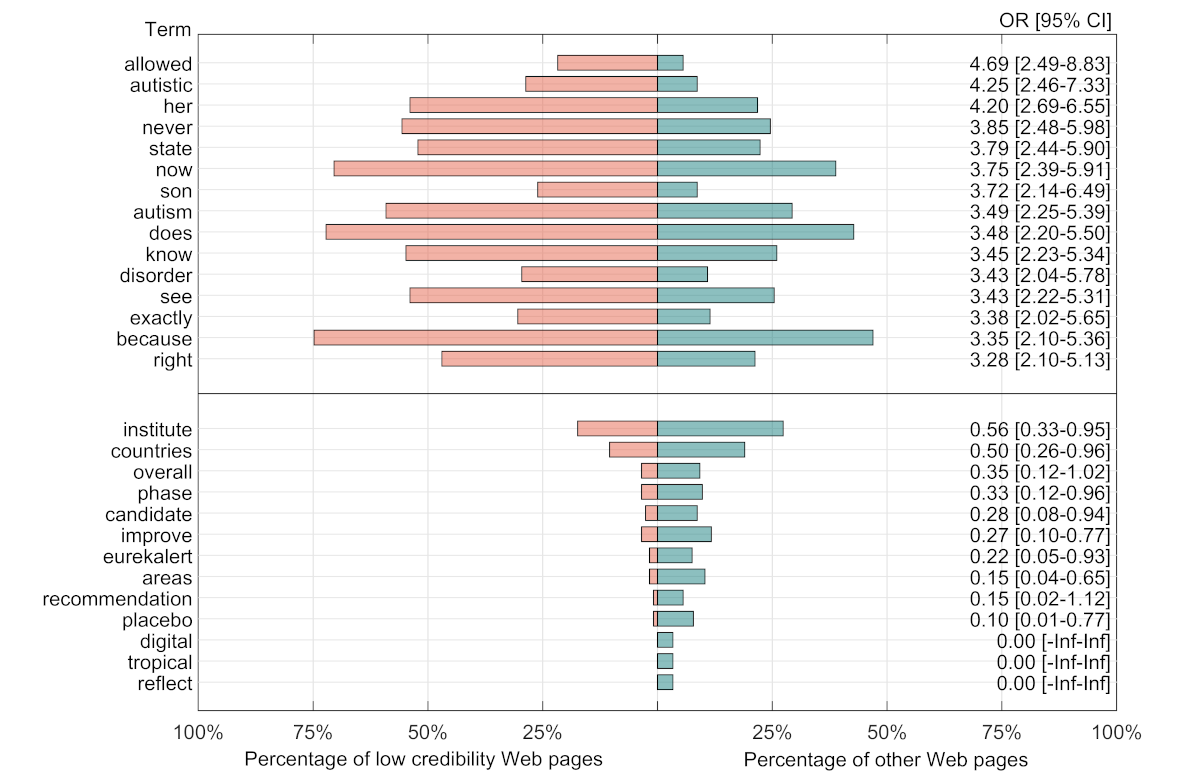
A subset of the terms that were informative of low-credibility scores in the training set of 474 Web pages. Terms at the top are those most over-represented in low-credibility Web pages compared with other Web pages, and terms at the bottom are those most under-represented in low-credibility Web pages compared with other Web pages. OR: odds ratio; Inf: infinity.

To consider the expected robustness of the classifiers, we additionally analyzed the set of terms that were most informative of low-credibility Web pages. We used a Fisher exact test to compare the proportion of low-credibility Web pages a term appeared in at least once relative to the proportion of other Web pages in which the term appeared at least once, examining the terms that were over-represented in either direction (Figure 4). 

The results indicate a set of mostly general terms; terms that are most indicative of low-credibility Web pages are related to stories about individuals and individual autonomy (eg, “her,” “son,” “autistic,” “right,” and “allowed”), and terms that are most indicative of other Web pages are related to research and populations (eg, “institute,” “phase,” “placebo,” “countries,” “improve,” and “tropical”). The results suggest that the sample of Web pages used to construct the training data is a broad enough sample to capture general patterns rather than specific repeated topics that would limit the external validity of the approach.

**Table 3:** Performance of the classifiers (average F_1_ score and accuracy in 10-fold cross-validation).

Criterion	Deep learning^a^, mean (SD)		Support vector machines^a^, mean (SD)		Random forests^a^, mean (SD)	
	F_1_ score	Accuracy	F_1_ score	Accuracy	F_1_ score	Accuracy
1	0.851 (0.005)	0.740 (0.008)	0.903 (0.032)	0.842 (0.045)	*0.950* (0.015)	0.924 (0.019)
2	0.000 (0.000)	0.638 (0.003)	0.802 (0.044)	0.828 (0.018)	*0.915* (0.005)	0.943 (0.006)
3	0.000 (0.000)	0.865 (0.009)	*0.761* (0.038)	0.917 (0.011)	0.745 (0.088)	0.944 (0.018)
4	0.882 (0.001)	0.789 (0.002)	0.903 (0.042)	0.833 (0.068)	*0.959* (0.017)	0.936 (0.022)
5	0.551 (0.249)	0.486 (0.051)	0.787 (0.034)	0.721 (0.051)	*0.921* (0.022)	0.920 (0.020)
6	0.867 (0.002)	0.765 (0.004)	0.912 (0.006)	0.852 (0.010)	*0.964* (0.002)	0.943 (0.004)
7	0.000 (0.000)	0.840 (0.008)	*0.801* (0.029)	0.924 (0.006)	0.764 (0.057)	0.936 (0.004)

^a^The classifier with the highest F1-score is italicized for each criterion.

### Potential Exposure Estimation

**Figure 5:**
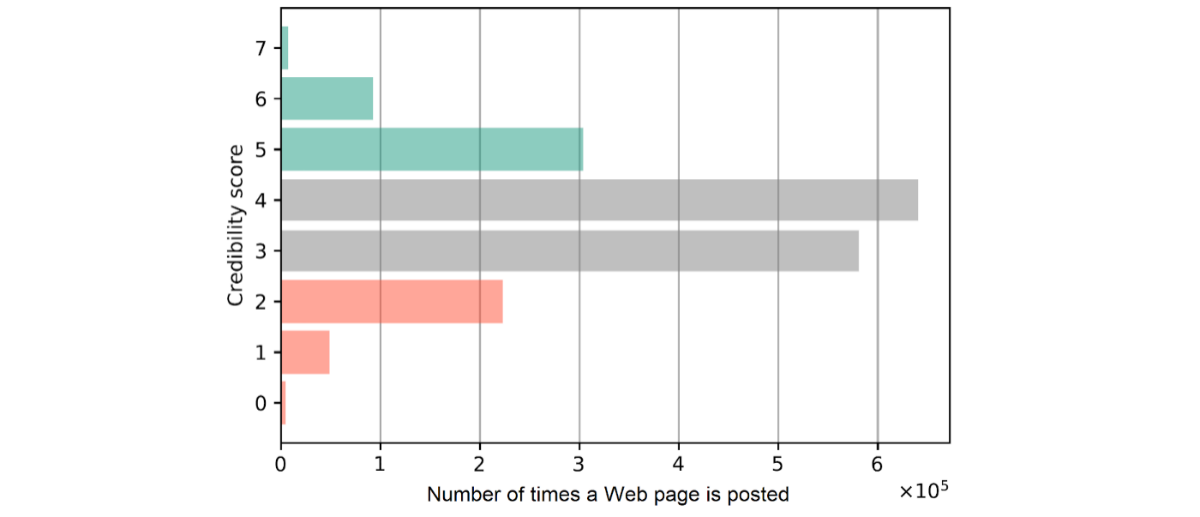
The sum of tweets and retweets for links to included Web pages relative to the number of credibility criteria satisfied.

Satisfied with the performance of the ensemble classifier, we then applied it to the full set of 144,003 unique vaccine-related Web pages, producing an estimated credibility score for every page. Fewer Web pages with low-credibility scores were shared on Twitter relative to those with medium- or high-credibility scores (Figure 5), although it is important to consider the performance limitations of the ensemble classifier when interpreting these findings. We estimated that 11.86% (16,961 of 143,003) of Web pages were of low credibility, and they generated 14.68% (112,225 of 764,283) of retweets. In comparison, 23.52% (33,636 of 143,003) of Web pages were of high credibility, and they generated 21.04% (160,777 of 764,283) of all retweets.

**Figure 6:**
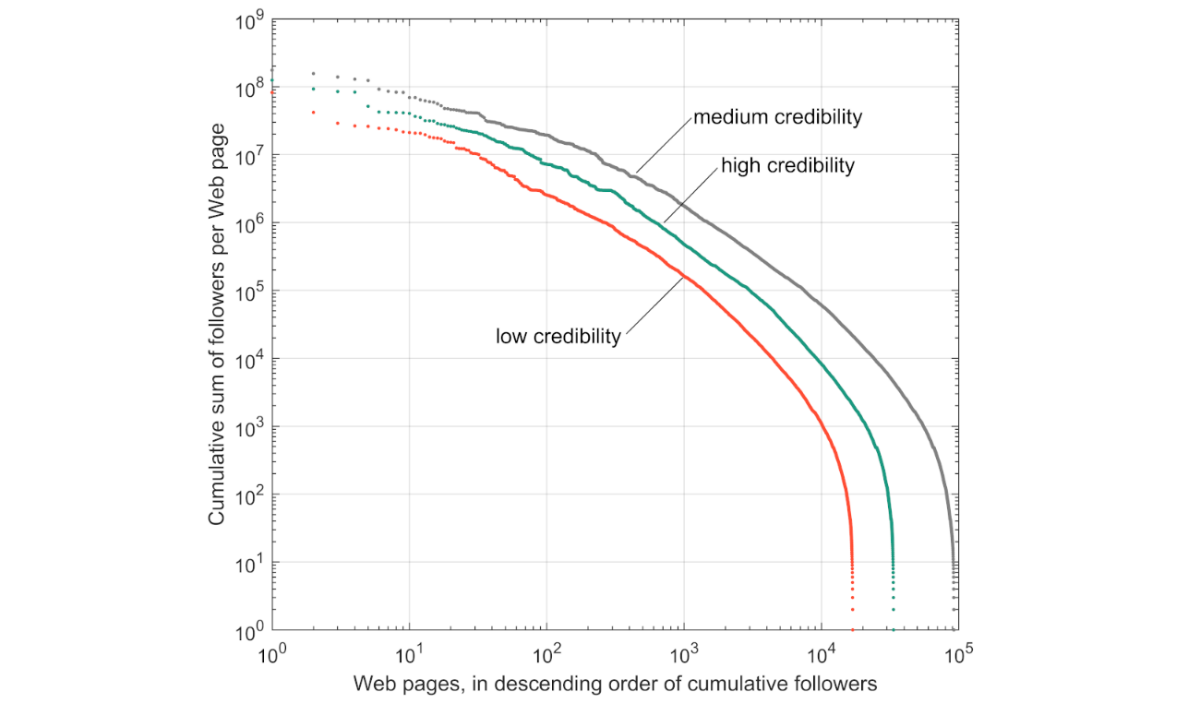
The distribution of potential exposures per Web page for low (orange), medium (gray), and high (cyan) credibility scores, where low credibility includes scores from 0 to 2, and high credibility includes scores from 5 to 7.

When we examined the total number of potential exposures by counting cumulative followers across all tweets and retweets for each Web page, we found that the distributions were similar (illustrated by the slopes of the 3 distributions in Figure 6).

Measured by the total proportion of exposures to links to relevant Web pages, tweets to low credibility Web pages produced 9.34% (1.64 billion of 17.6 billion) of total exposures, compared with the 24.59% (4.33 billion of 17.6 billion) of total exposures to high-credibility Web pages. This indicates that Twitter users sharing links to high-credibility and medium-credibility vaccine-related Web pages tended to have a greater number of followers than those sharing links to low-credibility vaccine-related Web pages. However, the shape of the distribution shows that some of the low-credibility Web pages may have been influential; the top 100 Web pages by exposure were included in tweets that may have been seen by 2 million to 80 million users, and more than 200 Web pages of low credibility were included in tweets that could have reached 1 million users.

**Figure 7:**
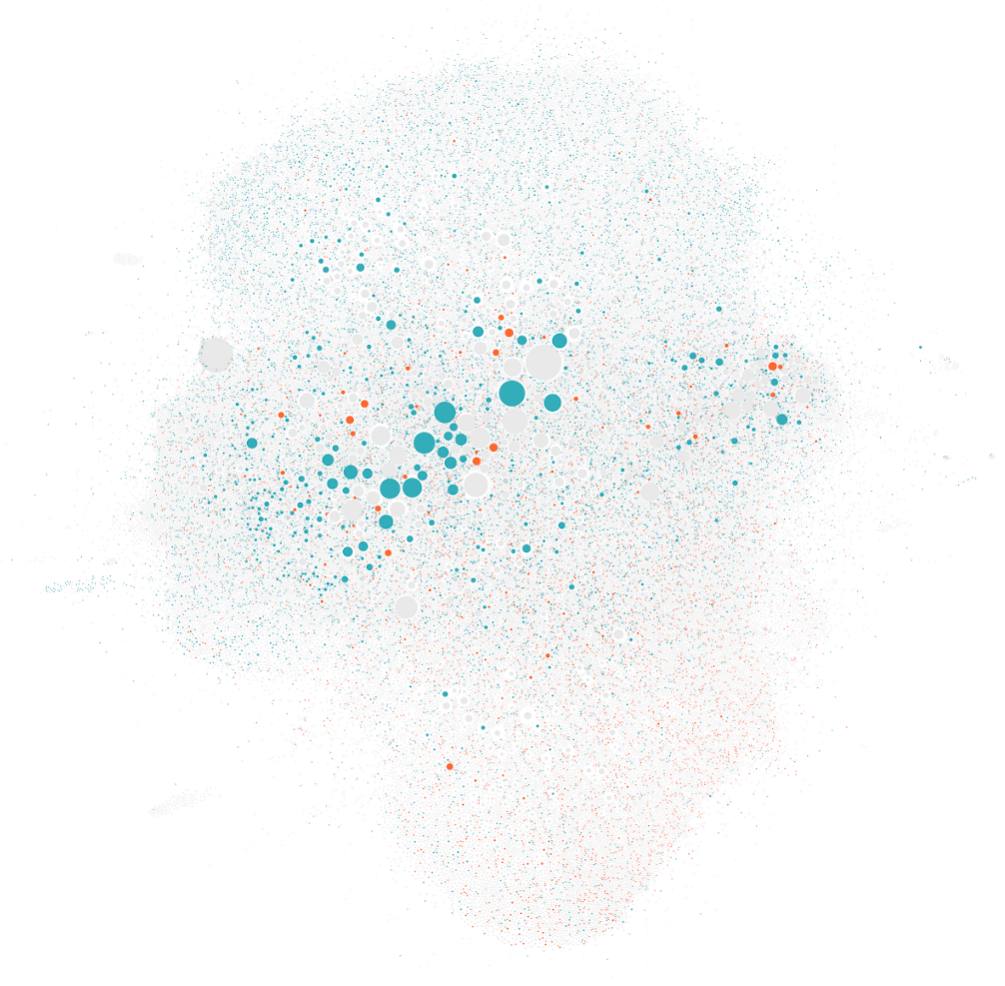
A network visualization representing the subset of 98,663 Twitter users who posted tweets including links to vaccine-related Web pages at least twice and were connected to at least one other user in the largest connected component. Users who posted at least 2 high-credibility Web pages and no low-credibility Web pages (cyan) and those who posted at least two low-credibility Web pages and no high-credibility Web pages (orange) are highlighted. The size of the nodes is proportional to the number of followers each user has on Twitter, and nodes are positioned by a heuristic such that well-connected groups of users are more likely to be positioned close together in the network diagram.

Links to low-credibility vaccine-related Web pages were more heavily concentrated among certain groups of users posting tweets about vaccines on Twitter. This is evident in a visualization of the follower network for the set of 98,663 Twitter users who posted at least two links to Web pages included in the study (Figure 7). The network indicates heterogeneity in the sharing of links to low-credibility vaccine-related Web pages, suggesting that there are likely to be communities of social media users for whom the majority of what they see and read about vaccines is of low credibility.

## Discussion

### Principal Findings

We found that it is feasible to produce machine learning classifiers to identify vaccine-related Web pages of low credibility. Applying a classifier to vaccine-related Web pages shared on Twitter between January 2017 and March 2018, we found that fewer low-credibility Web pages were shared overall, though some had a potential reach of tens of millions of Twitter users. A network visualization suggested that certain communities of Twitter users were much more likely to share and be exposed to low-credibility Web pages.

### Research in Context

This research extends knowledge related to the surveillance of health misinformation on social media. Where much of the prior research has aimed to label individual social media posts or the claims made on social media by veracity [25-29], we instead labeled Web pages shared on social media using a credibility appraisal checklist extended from previously validated instruments to be appropriate to vaccine-related communications [21,22]. In other related work, Mitra et al [51] examined the linguistic features in social media posts that influenced perceptions of credibility. Although we did not examine the linguistic features of the tweets that included links to low-credibility information, it would be interesting to connect these ideas to better understand whether they influence user behavior—making users more likely to engage with a tweet by URL access, replying, and sharing.

The work presented here is also different from previous studies examining opinions and attitudes expressed by Twitter users, which mostly label individual tweets or users based on whether they are promoting vaccination or advocating against vaccines [30,32,35,38]. Here we consider the communications shared on Twitter rather than the opinions expressed by users in the text of tweets.

Our study is also not directly comparable with previous studies that have examined how misinformation spreads through social media [2-6]. We examined a single topic that might not generalize to other application domains such as politics, labeled information according to a broader set of criteria than just the veracity of the information, and measured total potential exposures rather than just cascades of tweets and retweets. Rather than sampling from a set of known examples of fake and real news to compare spread, we sampled from across the spectrum of relevant articles shared on Twitter. Structuring the experiments in this way, we found no clear difference in the distribution of total potential exposures between low-credibility Web pages and others. Although most low-credibility Web pages are shared with a smaller number of Twitter users, some had the potential to reach tens of millions.

### Implications

This study has implications for public health. The ability to measure how people engage with and share misinformation on social media may help us better target and monitor the impact of public health interventions [39-41]. We found that certain subpopulations of Twitter users share low-credibility vaccine communications more often and are less likely to be connected to users sharing higher-credibility vaccine communications. Although these results are unsurprising, most studies examining vaccines on social media have only counted tweets rather than examining the heterogeneity of potential exposure to vaccine critical posts [30,38,52], despite evidence of the clustering of opinions from as early as 2011 [32]. This study is consistent with these previous findings on clustering and studies examining exposure to different topics about HPV vaccines [35,37]. Knowing where low-credibility communications are most commonly shared on social media may support the development of communication interventions targeted specifically at communities that are most likely to benefit [53]. Although the methods are not yet precise enough to reliably identify individual links to low-credibility communications, they may eventually be useful as the basis for countermeasures such as active debunking. Methods for inoculating against misinformation by providing warnings immediately before access have mixed results [10,54,55].

### Limitations

There were several limitations to this study. Although we used a modified sampling strategy to ensure a more balanced representation of Web pages, the manually labeled sample used for training and internal validation was relatively small, and this might have affected the results in 2 ways. First, our results showed that the DL-based classifiers were less accurate than the RF-based classifiers, but this might have been the consequence of the available training data rather than the general value of the DL approach. Without testing on larger sets of training data, we are unable to reliably conclude about the comparative performance of the machine learning methods. Second, in some document classification tasks where features are relatively sparse or many documents are very similar, using a smaller set of labeled examples can lead to overfitting. To avoid this, we were careful about removing duplicates and Web pages with overlapping text.

A second type of limitation relates to the choices we made about the methods. Other methods and architectures could have been used to predict credibility from text. For example, we could have used simpler methods including Naïve Bayes and logistic regression, used a single multi-label classifier to predict whether a document extracted from a Web page satisfied any of the criteria, or constructed a model that directly predicts the credibility score rather than the individual components.

A further limitation relates to the external validity of the classifier and our inability to draw conclusions about Web pages that do not include contiguous sections of text. We included only Web pages from which we could extract contiguous blocks of text and used a novel approach to sampling from those Web pages to create a reasonably balanced sample across the set of credibility scores. Other URLs included in vaccine-related tweets included links to other social media posts (including links to other tweets), links to YouTube and Instagram, links to memes in which text is embedded in an image, links to dynamic pages that no longer show the same information, and links to a range of other pages that included videos or images alongside a small amount of text. As we were unable to estimate the credibility of the vaccine-related information presented on these other Web pages, our conclusions are limited to the characterization of text-based Web pages. It is likely that a substantial proportion of Instagram, Facebook, and YouTube Web pages would receive a low-credibility score if they were assessed [56-58], which means we may have underestimated the sharing of low-credibility vaccine-related communications on Twitter.

Our estimates of exposure were imperfect. To estimate how many Twitter users might have been exposed to information relative to credibility, we summed the total number of followers of a user for each user that posted the link. We did not count the total number of unique followers who might have seen the link, did not report the number of likes, and do not have access to the number of replies. In the absence of more detailed measures of engagement that can estimate the number of times a Web page was accessed via Twitter, we felt measures of potential exposure were a reasonable upper bound. The conclusions related to measures of potential exposure, therefore, need to be interpreted with caution, and further studies using robust epidemiological designs are needed to reliably estimate exposure.

### Conclusions

We developed and tested machine learning methods to support the automatic credibility appraisal of vaccine-related information on the Web, showing that it is feasible. This allowed us to scale our analysis of large-scale patterns of potential exposure to low-credibility vaccine-related Web pages shared on Twitter. We found that although low-credibility Web pages were shared less often overall, there were certain subpopulations where the sharing of low-credibility Web pages was common. The results suggest two new ways to address the challenge of misinformation, including ongoing surveillance to identify at-risk communities and better target resources in health promotion and embedding the tool in interventions that flag low-credibility communications for consumers as they engage with links to Web pages on social media.

## Abbreviations

DL: deep learning
HPV: human papillomavirus
QIMR: Quality Index for health-related Media Reports
RF: random forests
RNN: recurrent neural networks
SVM: support vector machines
tf-idf: term frequency–inverse document frequency

## References

[1] Lazer DM, Baum MA, Benkler Y, Berinsky AJ, Greenhill KM, Menczer F, Metzger MJ, Nyhan B, Pennycook G, Rothschild D, Schudson M, Sloman SA, Sunstein CR, Thorson EA, Watts DJ, Zittrain JL (2018). The science of fake news. Science.

[2] Budak C, Agrawal D, El AA (2011). Limiting the Spread of Misinformation in Social Networks. Proceedings of the 20th International Conference on World Wide Web.

[3] Mocanu D, Rossi L, Zhang Q, Karsai M, Quattrociocchi W (2015). Collective attention in the age of (mis)information. Comput Hum Behav.

[4] Tambuscio M, Ruffo G, Flammini A, Menczer F (2015). Fact-Checking Effect on Viral Hoaxes: A Model of Misinformation Spread in Social Networks. Proceedings of the 24th International Conference on World Wide Web.

[5] Kumar S, West R, Leskovec J (2016). Disinformation on the Web: Impact, Characteristics, and Detection of Wikipedia Hoaxes. Proceedings of the 25th International Conference on World Wide Web.

[6] Vosoughi S, Roy D, Aral S (2018). The spread of true and false news online. Science.

[7] Grinberg N, Joseph K, Friedland L, Swire-Thompson B, Lazer D (2019). Fake news on Twitter during the 2016 US presidential election. Science.

[8] del Vicario M, Bessi A, Zollo F, Petroni F, Scala A, Caldarelli G, Stanley HE, Quattrociocchi W (2016). The spreading of misinformation online. Proc Natl Acad Sci U S A.

[9] Weaver JB, Thompson NJ, Weaver SS, Hopkins GL (2009). Healthcare non-adherence decisions and internet health information. Comput Hum Behav.

[10] Lewandowsky S, Ecker UK, Seifert CM, Schwarz N, Cook J (2012). Misinformation and its correction: continued influence and successful debiasing. Psychol Sci Public Interest.

[11] Schwitzer G (2002). A review of features in internet consumer health decision-support tools. J Med Internet Res.

[12] Eysenbach G, Köhler C (2002). How do consumers search for and appraise health information on the world wide web? Qualitative study using focus groups, usability tests, and in-depth interviews. Br Med J.

[13] (2019). Reuters Institute Digital News Report.

[14] Winters M, Larsson A, Kowalski J, Sundberg CJ (2019). The association between quality measures of medical university press releases and their corresponding news stories-important information missing. PLoS One.

[15] Yavchitz A, Boutron I, Bafeta A, Marroun I, Charles P, Mantz J, Ravaud P (2012). Misrepresentation of randomized controlled trials in press releases and news coverage: a cohort study. PLoS Med.

[16] Haneef R, Ravaud P, Baron G, Ghosn L, Boutron I (2017). Factors associated with online media attention to research: a cohort study of articles evaluating cancer treatments. Res Integr Peer Rev.

[17] Sumner P, Vivian-Griffiths S, Boivin J, Williams A, Venetis CA, Davies A, Ogden J, Whelan L, Hughes B, Dalton B, Boy F, Chambers CD (2014). The association between exaggeration in health related science news and academic press releases: retrospective observational study. Br Med J.

[18] Grundy Q, Dunn AG, Bourgeois FT, Coiera E, Bero L (2018). Prevalence of disclosed conflicts of interest in biomedical research and associations with journal impact factors and altmetric scores. J Am Med Assoc.

[19] Bernstam EV, Shelton DM, Walji M, Meric-Bernstam F (2005). Instruments to assess the quality of health information on the World Wide Web: what can our patients actually use?. Int J Med Inform.

[20] Steffens M, Dunn A, Leask J (2017). Meeting the challenges of reporting on public health in the new media landscape. Aust J Rev.

[21] Charnock D, Shepperd S, Needham G, Gann R (1999). DISCERN: an instrument for judging the quality of written consumer health information on treatment choices. J Epidemiol Community Health.

[22] Zeraatkar D, Obeda M, Ginsberg JS, Hirsh J (2017). The development and validation of an instrument to measure the quality of health research reports in the lay media. BMC Public Health.

[23] HealthNewsReview.

[24] Larson H (2018). The biggest pandemic risk? Viral misinformation. Nature.

[25] Qazvinian V, Rosengren E, Radev D, Mei Q (2011). Rumor Has It: Identifying Misinformation in Microblogs. Proceedings of the Conference on Empirical Methods in Natural Language Processing.

[26] Zhao Z, Resnick P, Mei Q (2015). Enquiring Minds: Early Detection of Rumors in Social Media from Enquiry Posts. Proceedings of the 24th International Conference on World Wide Web.

[27] Vosoughi S, Mohsenvand M, Roy D (2017). Rumor gauge: predicting the veracity of rumors on Twitter. ACM Trans Knowl Discov Data.

[28] Ma J, Gao W, Mitra P, Kwon S, Jansen BJ, Wong K-F, Cha M (2016). Detecting Rumors From Microblogs With Recurrent Neural Networks. Proceedings of the Twenty-Fifth International Joint Conference on Artificial Intelligence.

[29] Ruchansky N, Seo S, Liu Y (2017). CSI: A Hybrid Deep Model for Fake News Detection. Proceedings of the 2017 ACM on Conference on Information and Knowledge Management.

[30] Broniatowski DA, Jamison AM, Qi S, AlKulaib L, Chen T, Benton A, Quinn SC, Dredze M (2018). Weaponized health communication: Twitter bots and Russian trolls amplify the vaccine debate. Am J Public Health.

[31] Dredze M, Broniatowski DA, Smith MC, Hilyard KM (2016). Understanding vaccine refusal: why we need social media now. Am J Prev Med.

[32] Salathé M, Khandelwal S (2011). Assessing vaccination sentiments with online social media: implications for infectious disease dynamics and control. PLoS Comput Biol.

[33] Du J, Tang L, Xiang Y, Zhi D, Xu J, Song H, Tao C (2018). Public perception analysis of tweets during the 2015 measles outbreak: comparative study using convolutional neural network models. J Med Internet Res.

[34] Chew C, Eysenbach G (2010). Pandemics in the age of Twitter: content analysis of tweets during the 2009 H1N1 outbreak. PLoS One.

[35] Dunn AG, Leask J, Zhou X, Mandl KD, Coiera E (2015). Associations between exposure to and expression of negative opinions about human papillomavirus vaccines on social media: an observational study. J Med Internet Res.

[36] Dunn AG, Surian D, Leask J, Dey A, Mandl KD, Coiera E (2017). Mapping information exposure on social media to explain differences in HPV vaccine coverage in the United States. Vaccine.

[37] Surian D, Nguyen DQ, Kennedy G, Johnson M, Coiera E, Dunn AG (2016). Characterizing Twitter discussions about HPV vaccines using topic modeling and community detection. J Med Internet Res.

[38] Du J, Cunningham RM, Xiang Y, Li F, Jia Y, Boom JA, Myneni S, Bian J, Luo C, Chen Y, Tao C (2019). Leveraging deep learning to understand health beliefs about the Human Papillomavirus Vaccine from social media. NPJ Digit Med.

[39] Shao C, Ciampaglia G, Flammini A, Menczer F (2016). Hoaxy: A Platform for Tracking Online Misinformation. Proceedings of the 25th International Conference Companion on World Wide Web.

[40] Dunn AG, Mandl KD, Coiera E (2018). Social media interventions for precision public health: promises and risks. NPJ Digit Med.

[41] Colditz JB, Chu K, Emery SL, Larkin CR, James AE, Welling J, Primack BA (2018). Toward real-time infoveillance of Twitter health messages. Am J Public Health.

[42] Coiera E (1998). Information epidemics, economics, and immunity on the internet. We still know so little about the effect of information on public health. Br Med J.

[43] (2018). Google Code.

[44] (2009). Centers for Disease Control and Prevention.

[45] Moynihan R, Bero L, Ross-Degnan D, Henry D, Lee K, Watkins J, Mah C, Soumerai SB (2000). Coverage by the news media of the benefits and risks of medications. N Engl J Med.

[46] Association of Health Care Journalists.

[47] Wiggers J The University of Newcastle.

[48] Betsch C, Rossmann C, Habersaat KB, Pfeiffer D, Holtmann C, Korn L (2017). World Health Organization Europe.

[49] Merity S, Keskar NS, Socher R (2017). arXiv.

[50] Howard J, Ruder S (2018). Universal Language Model Fine-Tuning for Text Classification. Proceedings of the 56th Annual Meeting of the Association for Computational Linguistics.

[51] Mitra T, Wright G, Gilbert E (2017). A Parsimonious Language Model of Social Media Credibility Across Disparate Events. Proceedings of the 2017 ACM Conference on Computer Supported Cooperative Work and Social Computing.

[52] Tomeny TS, Vargo CJ, El-Toukhy S (2017). Geographic and demographic correlates of autism-related anti-vaccine beliefs on Twitter, 2009-15. Soc Sci Med.

[53] Vraga EK, Bode L (2017). Using expert sources to correct health misinformation in social media. J Sci Commun.

[54] Bode L, Vraga EK (2018). See something, say something: correction of global health misinformation on social media. Health Commun.

[55] Pennycook G, Cannon TD, Rand DG (2018). Prior exposure increases perceived accuracy of fake news. J Exp Psychol Gen.

[56] Briones R, Nan X, Madden K, Waks L (2012). When vaccines go viral: an analysis of HPV vaccine coverage on YouTube. Health Commun.

[57] Madathil KC, Rivera-Rodriguez AJ, Greenstein JS, Gramopadhye AK (2015). Healthcare information on YouTube: a systematic review. Health Informatics J.

[58] Venkatraman A, Garg N, Kumar N (2015). Greater freedom of speech on web 2.0 correlates with dominance of views linking vaccines to autism. Vaccine.

